# First records of the uristid lysianassoids from Korean waters: redescription of *Anonyx abei* Takekawa & Ishimaru, 2001 and description of *Anonyx exilipes* sp. n. (Crustacea, Amphipoda, Uristidae)

**DOI:** 10.3897/zookeys.733.22021

**Published:** 2018-01-31

**Authors:** Tae Won Jung, Charles Oliver Coleman, Ji Hyung Kim, Seong Myeong Yoon

**Affiliations:** 1 Museum für Naturkunde Berlin, 10115 Berlin, Germany; 2 Infectious Disease Research Center, Korea Research Institute of Bioscience and Biotechnology, Daejeon 34141, South Korea; 3 Department of Biology, Chosun University, Gwangju 61452, South Korea

**Keywords:** Amphipods, *Anonyx abei*, *Anonyx exilipes* sp. n., Korea, taxonomy, uristids

## Abstract

The uristid lysianassoids are reported for the first time from Korean waters with a redescription of *Anonyx abei* Takekawa & Ishimaru, 2001 and the description of *Anonyx exilipes* **sp. n.** *Anonyx abei* is characterized by a distinctively small projection of the posterodistal corner on epimeron 3, different from all species of the *Anonyx nugax* group which share a constriction at the point of insertion of a distal seta on the inner ramus of uropod 2. *Anonyx exilipes* **sp. n.** is included in the *Anonyx laticoxae* group characterized by the unconstricted inner ramus of uropod 2. This new species is distinguished from other *A. laticoxae* group species by the longer and more slender carpus and propodus of pereopod 6, and the non-lobate merus of pereopod 7.

## Introduction

The family Uristidae Hurley, 1963 is a widespread and large group of lysianassoid amphipods containing more than 180 species in 25 well-defined genera (Lowry and Kilgallen 2014, WoRMS 2017). This family is characterized by the modified mouthparts in most species such that the apical setae of the outer lobe of maxilla 1 show a 7/4 arrangement, the mandibular incisor forms a curved blade, the molar is modified into a setose tongue, and the inner lobe of maxilla 2 is significantly shorter than the outer lobe (Lowry and Stoddart 1989, 1997, Lowry and Kilgallen 2014).

*Anonyx* Krøyer, 1838 is one of the largest genera of uristids constituted of about 50 species described from the arctic-boreal region (Takekawa and Ishimaru 2001, Lowry and Kilgallen 2014, WoRMS 2017). Steele (1979, 1982, 1983, 1986, 1989, 1991) studied on this genus extensively and divided it into five informal subgroups based on the states of a constriction at the inner ramus on uropod 2: 1) the *A. laticoxae* group: ramus without a constriction at the insertion of distal robust seta, which equal to or slightly longer than proximal seta; 2) the *A. validus* group: uropod 2 or inner ramus expanded laterally, lacking a constriction, setae small or lacking; 3) the *A. nugax* group: inner ramus with a constriction at the point of insertion of a distal seta, which longer than proximal setae; 4) the *A. compactus* group: inner ramus constricted beyond the point of insertion of the distal seta which much longer than proximal setae; and 5) the *A. bispinosus* group: inner ramus completely constricted beyond the point of insertion of the distal seta, and the proximal portion of the inner ramus laterally flattened. Until now, it is not certain if these taxonomic groupings of Steele are reflecting the phylogeny. Nevertheless, these groups are useful for identification of the *Anonyx* species, because the states of constriction at the inner ramus on uropod 2 can easily be observed.

In spite of the species abundance and wide range of distribution, the taxonomic study on lysianassoids is insufficient in Korea and only eight species have been reported: *Aroui minusetosus* Jung, Coleman & Yoon, 2017; *Lepidepecreum vitjazi* Gurjanova, 1962; *Orchomenella japonica* Gurjanova, 1962; *Orchomenella obtusa* (GO Sars, 1891); *Orchomenella paucisetigera* Jung, Yi, Coleman & Yoon, 2017; *Orchomenella rugosa* Jung, Yi, Coleman & Yoon, 2017; *Pseudorchomene boreoplebs* Jung, Coleman & Yoon, 2017; and *Socarnes tongyeongensis* Kim & Hendrycks, 2013 (The Korean Society of Systematic Zoology 1997, Jung and Kim 2008, Kim and Hendrycks 2013, Jung et al. 2015, 2017a, 2017b, 2017c). However, none of these are members of the Uristidae. This is the first record of the family Uristidae from Korean waters.

### Materials and methods

Collected specimens were initially fixed in 80% ethyl alcohol in the field and then preserved in 95% ethyl alcohol after sorting in the laboratory. Specimens were stained with lignin pink before dissection. Their appendages were dissected in petri dishes or on excavated microscopic slides filled with glycerol using forceps and needles under a stereomicroscope (Leica M205). They were mounted onto temporary slides using glycerol-ethanol mixed solution or permanent slides using polyvinyl lactophenol solution. For making illustrations, pencil drawings were performed under a light microscope (Leica DMLB) with the aid of a drawing tube. Drawings were scanned, inked digitally and arranged to plates using the methods described in Coleman (2003, 2009). Examined materials are deposited at the National Institute of Biological Resources (**NIBR**) of Korea.

## Systematic accounts

### Order Amphipoda Latreille, 1816

#### Superfamily Lysianassoidea Dana, 1849

##### Family Uristidae Hurley, 1963

Korean name: Na-do-gin-pal-yeop-sae-u-gwa, new

###### Genus *Anonyx* Krøyer, 1838

Korean name: Na-do-gin-pal-yeop-sae-u-sok, new

####### Anonyx abei

Taxon classificationAnimaliaAmphipodaUristidae

Takekawa & Ishimaru, 2001

Figs 1 , 2 , 3 , 4

Anonyx abei Takekawa & Ishimaru, 2001: 410, figs 6–10.

######## Material examined.

One male (9.3 mm) and one female (7.8 mm), NIBRV0000807162, Korea: Jeju-do, Beom Is., 33°12.9945N 126°32.215E, depth 66 m, 1 Nov 2016, collected by a light trap.

######## Diagnosis.

Gnathopod 1 subchelate; basis weakly setose anteriorly; propodus posterior margin forming weak lobe together palm posterodistally, palm serrated, defined by one pair of elongate robust setae; dactylus with strong protrusion on inner margin. Gnathopod 2 minutely chelate; propodus subquadrate, with nine robust setae anterodistally, posterodistal corner produced distally with two robust setae, palm short, with small cavity; dactylus anchored at middle of distal margin on propodus, inner margin denticulate. Epimeron 2 posteroventral corner a little produced. Uropod 2 inner and outer rami each with a constriction at insertion point of distal elongate seta on dorsal surface.

######## Description of male.

**Figure 1.**
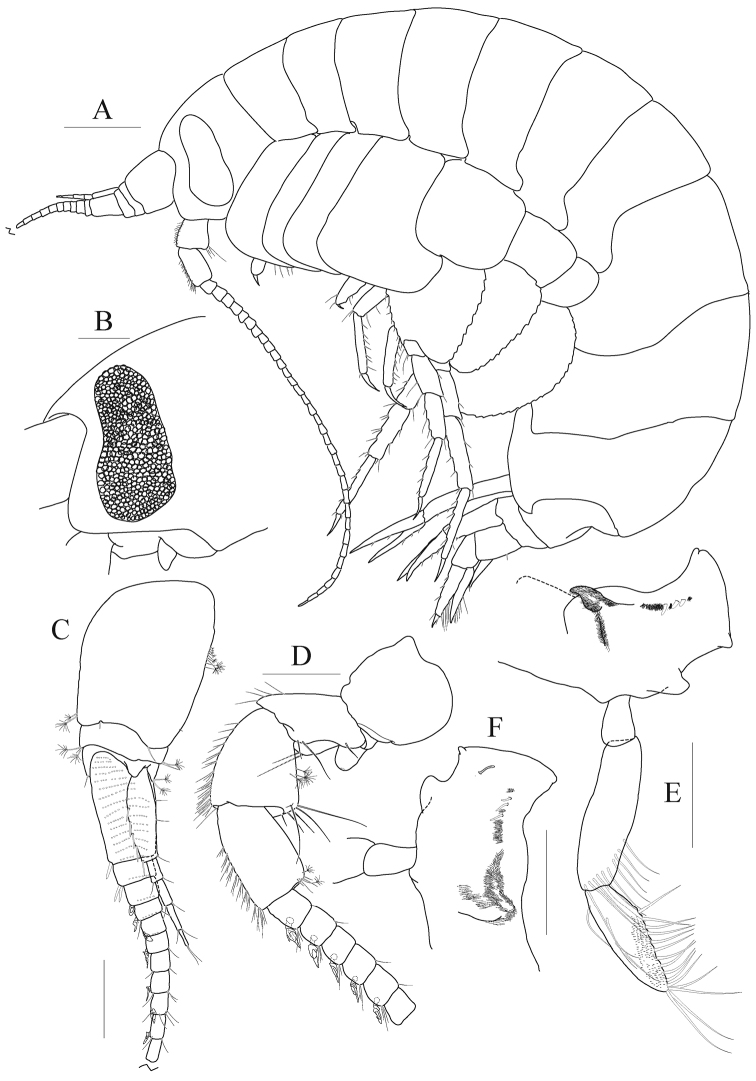
*Anonyx abei* Takekawa & Ishimaru, 2001, male, NIBRV0000807162, 9.3 mm. **A** habitus **B** head **C** antenna 1, medial **D** antenna 2 **E** right mandible **F** left mandible. Scale bars: 0.2 mm (**B–E**), 0.5 mm (**A**).

***Head*** (Fig. 1B). Lateral cephalic lobes expanded anteriorly, subtriangular, apex rounded; eye large, pyriform, occupying most of anterior part of head, composed of numerous small ommatidia.

*Antenna 1* (Fig. 1C) distinctly shorter than antenna 2; peduncle 1^st^ article largest, weakly expanded; accessory flagellum composed of five articles, 1^st^ article longest, with five robust setae on posterior margin; flagellum 1^st^ article distinctly elongate, with one robust seta at posterodistal corner, 2^nd^ article with one pair of robust setae at posterodistal corner, calceoli present from 3^rd^ article.

*Antenna 2* (Fig. 1A, D) elongate, 0.4 × as long as body; gland cone developed but apex blunt; peduncle 4^th^, 5^th^ articles expanded distally, setose on anterior margin; flagellum composed of 34 articles; calceoli present anterodistally.

*Mandible* (Fig. 1E, F) incisor smooth, but bearing blunt denticles on both sides; lacinia mobilis present on left side only, narrowly cylindrical (finger-like), slender; three small raker setae and a patch of short setules present between raker setae and molar processes; molar process not triturative, flap-shaped, densely pubescent, lateral setigerous crest present; palp composed of three articles, attached nearly at level of molar process; 2^nd^ article longest, with an oblique row of ten setae distally; 3^rd^ article falcate, 0.8 × as long as 2^nd^ article, inner margin lined with setae, apex with four setae.

**Figure 2.**
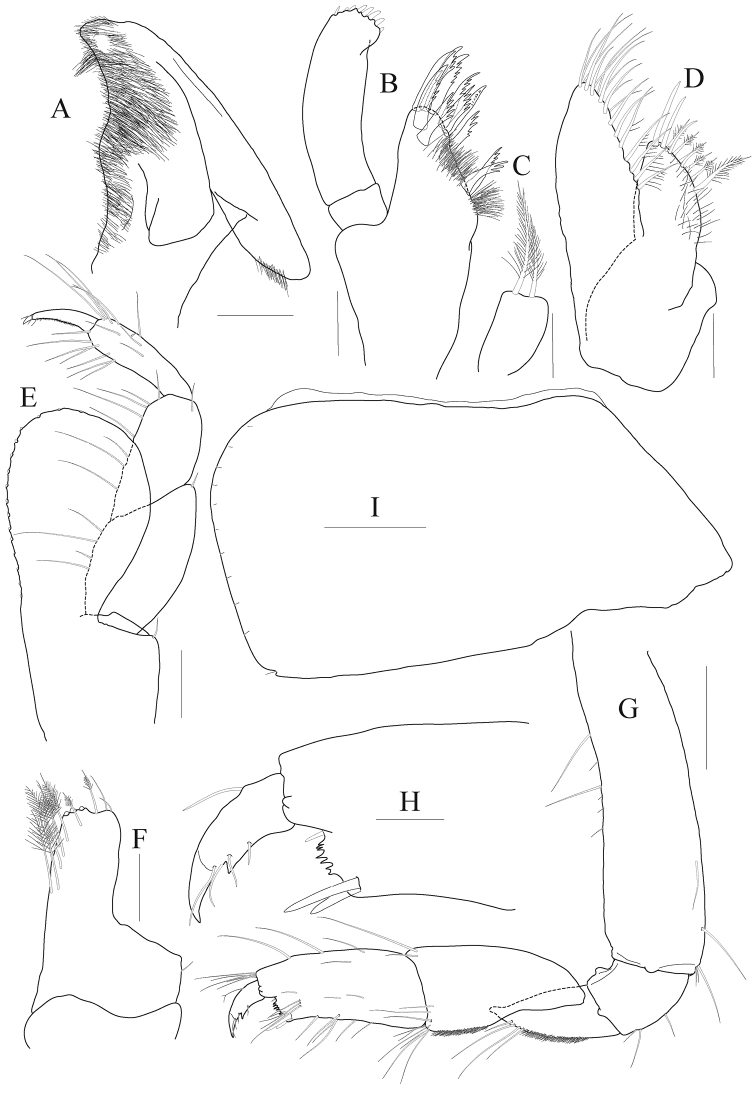
*Anonyx abei* Takekawa & Ishimaru, 2001, male, NIBRV0000807162, 9.3 mm. **A** lower lip **B** maxilla 1 **C** maxilla 1 inner lobe **D** maxilla 2 **E** maxilliped **F** maxilliped inner lobe **G** gnathopod 1 **H** gnathopod 1 palm and dactylus, enlarged **I** coxa 1. Scale bars: 0.05 mm (**H**), 0.1 mm (**A–F**), 0.2 mm (**G, I**).

*Lower lip* (Fig. 2A) densely pubescent; inner lobe indistinct.

*Maxilla 1* (Fig. 2B, C) inner lobe short, subquadrate distally, with two plumose setae on blunt apex; outer lobe with eleven toothed setae in 7/4 arrangement; palp bi-articulate, distal article width steady, slightly curved, with eight robust setae on apical margin.

*Maxilla 2* (Fig. 2D) inner lobe reduced, half as long as outer lobe, narrowing distally, with two rows of simple and plumose setae on mediodistal margin (proximal plumose seta longest); outer lobe also narrowing distally and with two setal rows on mediodistal margin.

*Maxilliped* (Fig. 2E, F) inner lobe subrectangular with one mediodistal row of plumose setae, apex blunt with three nodular setae; outer lobe well developed, subovoid, not beyond palp 3^rd^ article, lined with 16 nodular setae on mediodistal margin (all nodular setae small); palp composed of four articles, 1^st^ article expanded, 2^nd^ article with setae medially, 3^rd^ article slender, 4^th^ article half as long as article 3, apical seta robust, short.

***Pereon.*** *Gnathopod 1* (Fig. 2G–I) subchelate; coxa large, subquadrate, slightly expanded anteroventrally, with one small notch at posteroventral corner; basis 0.7 × as long as coxa, width nearly steady, anterior margin a little lobate distally, weakly setose; ischium moderate, with one small anterior lobe; merus triangular, 0.4 × as long as basis; carpus half as long as basis, convex anteroproximally, carpal lobe blunt, lined with minute setae; propodus 0.9 × as long as carpus, gradually diminished distally but forming weak lobe together palm posterodistally, palm distinct, serrated, defined by one pair of elongate robust setae; dactylus falcate, exceeding palm, with strong protrusion on inner margin.

**Figure 3.**
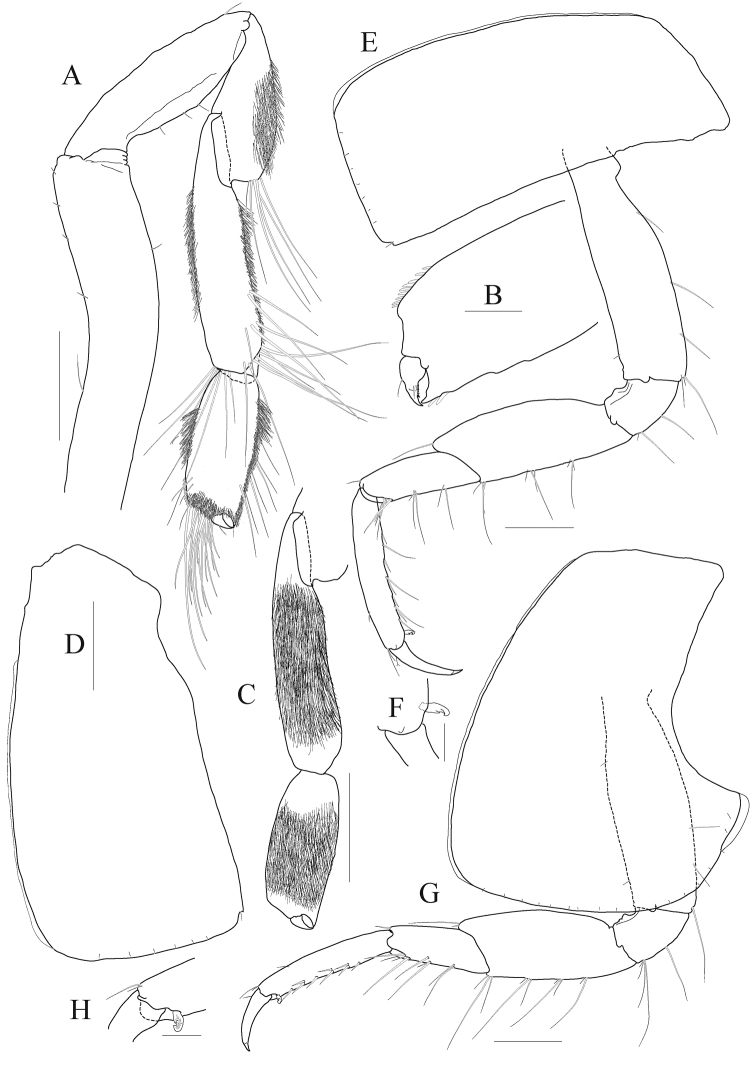
*Anonyx abei* Takekawa & Ishimaru, 2001, male, NIBRV0000807162, 9.3 mm. **A** gnathopod 2 **B** gnathopod 2 plam and dactylus **C** gnathopod 2 carpus to dactylus, medial **D** coxa 2 **E** pereopod 3 **F** pereopod 3 locking seta **G** pereopod 4 **H** pereopod 4 locking seta. Scale bars: 0.05 mm (**B, F, H**), 0.2 mm (**A, C–E, G**).

*Gnathopod 2* (Fig. 3A–D) slender, minutely chelate; coxa subrectangular, slightly divergent ventrally, with one small notch posteroventrally; basis as long as coxa, curved in midway; ischium elongate, 0.6 × as long as basis; merus 0.7 × as long as ischium, with numerous short setae posteriorly, posterodistal corner angular with many elongate setae; carpus 0.7 × as long as basis, margins and medial surface covered with numerous short setae, with elongate setae anteriodistally (longest seta exceeding propodus), posterior margin distal half also with elongate setae; propodus subquadrate, with nine robust setae anterodistally, posterodistal corner produced distally with two robust setae, palm short, with small cavity; dactylus short, anchored at middle of distal margin on propodus, inner margin denticulate, apex slightly exceeding corner of palm.

*Pereopod 3* (Fig. 3E, F) coxa subrectangular, 0.4 × as wide as long, with one small notch posteroventrally; basis 0.6 × as long as coxa, somewhat expanded posterodistally; ischium moderate size, with one small anterior lobe; merus expanded anteriorly, slightly tipped anterodistally; carpus 0.6 × as long as merus, not expanded; propodus 1.5 × as long as carpus, lined with simple setae on posterior margin, with one hooked locking seta posterodistally; dactylus falcate, elongate, 0.4 × as long as propodus, unguis weak.

*Pereopod 4* (Fig. 3G, H) coxa deeper than wide, expanded posteroventrally; other articles nearly similar with those of pereopod 3.

**Figure 4.**
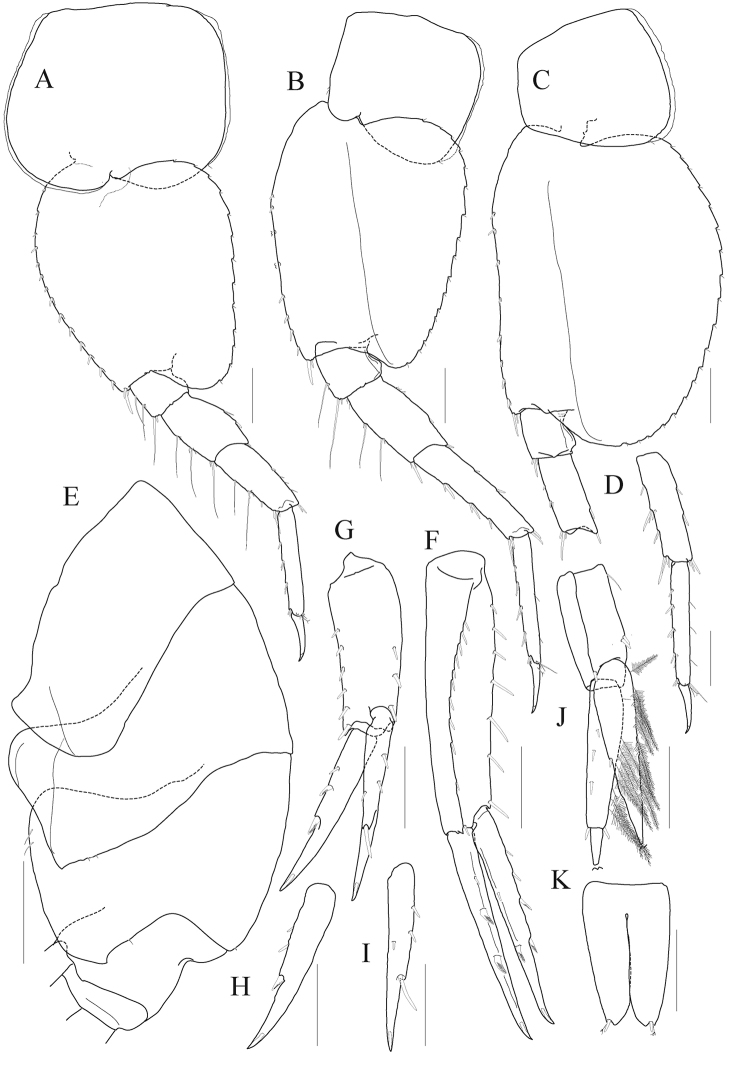
*Anonyx abei* Takekawa & Ishimaru, 2001, male, NIBRV0000807162, 9.3 mm. **A** pereopod 5 **B** pereopod 6 **C** pereopod 7 **D** pereopod 7 carpus to dactylus **E** pleonal epimera 1–3, lateral **F** uropod 1 **G** uropod 2 **H** uropod 2 outer ramus **I** uropod 2 inner ramus **J** uropod 3 **K** telson. Scale bars: 0.2mm (**A-D, F–K**), 0.5 mm (**E**).

*Pereopod 5* (Fig. 4A) coxa large, subquadrate, 1.2 × as wide as long, equilobate; basis subovoid, anterior margin rounded, lined with robust setae, with one pair of robust setae anterodistally (one seta elongate), posterior lobe well developed, more expanded proximally, margin somewhat flattened, crenulate, expanded posterodistal corner exceeding ischium; ischium to carpus lined with elongate slender setae and short setae anteriorly; merus expanded posteriorly; carpus subrectangular, narrowing distally, 1.2 × as long as merus; propodus linear, 1.3 × as long as carpus, lined with robust setae anteriorly, with one pair of locking setae; dactylus falcate, elongate, 0.4 × as long as propodus, unguis weak.

*Pereopod 6* (Fig. 4B) longer than pereopod 5; coxa subrectangular, smaller than that of pereopod 5, bilobate, anterior lobe small, posterior lobe more expanded posteroventrally; basis subovoid, 0.7 × as wide as long, 1.1 × as long as that of pereopod 5, anterior margin lined with robust setae on distal 2/3 margin, with one pair of robust setae anterodistally (one seta elongate), posterior lobe well developed, margin somewhat flattened, crenulate, expanded posterodistal corner not exceeding ischium; elongate slender setae present from ischium to merus anteriorly; merus subrectangular, 0.4 × as long as basis, a little expanded posteriorly, tipped posterodistally; carpus rectangular, not lobate, 1.3 × as long as merus, a little curved and slightly diminished distally; propodus slender, as long as carpus, lined with robust setae anteriorly, with one pair of locking setae; dactylus falcate, elongate, 0.4 × as long as propodus, unguis weak.

*Pereopod 7* (Fig. 4C, D) longer than pereopod 6; coxa unilobate, as large as that of pereopod 6, expanded posteroventrally; basis 1.2 × as wide and 1.1 × as long as that of pereopod 6, anterior margin slightly concaved at the middle, with robust setae on distal 3/4 margin, posterior lobe well developed, margin rounded, not flattened than those of pereopods 6–7; slender setae absent in ischium and merus; merus rectangular, not lobate, 0.3 × as long as basis, weakly produced posterodistally; carpus also rectangular, 1.2 × as long as merus; propodus slender, 1.1 × as long as carpus, lined with robust setae anteriorly, with one pair of locking setae; dactylus falcate, elongate, 0.4 × as long as propodus, unguis weak.

***Pleon.*** *Epimeron 1* weakly produced anteroventrally, rounded posteroventrally. *Epimeron 2* larger than epimeron 1, also produced anteroventrally, slightly convex ventrally, posteroventral corner a little produced. *Epimeron 3* largest, regularly rounded posteroventrally, posteroventral corner produced backwards. *Urosomite 1* with deep dorsal depression and distal carina weak (Fig. 4E).

*Uropod 1* (Fig. 4F) longest; peduncle 1.2 × as long as inner ramus, with eleven robust setae on dorsolateral margin and seven elongate robust setae on dorsomedial margin; rami subequal to each other; inner ramus with four dorsomedial and two dorsolateral robust setae (distal setae on both sides more robust and bearing wrinkly surfaces); outer ramus with three dorsolateral robust setae (distal two setae more robust and bearing wrinkly surfaces).

*Uropod 2* (Fig. 4G–I) 0.7 × as long as uropod 1; peduncle as long as inner ramus, with six robust setae on dorsolateral margin and three robust setae on dorsomedial margin; inner ramus 1.1 × as long as outer ramus, with one dorsolateral and two dorsomedial setae, with one constriction at insertion point of distal elongate seta on dorsal surface; outer ramus with three dorsolateral robust setae (with one constriction at insertion point of distal robust seta).

*Uropod 3* (Fig. 4J) 0.8 × as long as uropod 2; peduncle 0.7 × as long as inner ramus; both rami with plumose setae on medial margin; outer ramus bi-articulate, distal article 0.3 × as long as proximal article; inner ramus as long as inner ramus.

*Telson* (Fig. 4K) longer than broad, cleft to about 80%, each lobe with apical notch bearing one pair of robust seta and sensory seta.

######## Remarks.

The *Anonyx nugax* group of Steele (1982) is characterized by the presence of a constriction at the point of insertion of a distal seta which is longer than the proximal setae on the inner ramus of uropod 2. Takekawa and Ishimaru (2001) reported *Anonyx abei* as a new species from Japanese waters, and they assigned this species to the *Anonyx nugax* group based on the shape of the inner ramus on uropod 2, as mentioned above. *Anonyx abei* was differentiated from other 13 species included in the *A. nugax* group by the distinctively small projection of the posterodistal corner on epimeron 3 (Takekawa and Ishimaru 2001). As a result of the profound morphological examination, our Korean specimens are also show this character state and other characteristics also agree with the original description of Takekawa and Ishimaru (2001). However, there are some minor differences between the Korean and Japanese specimens: 1) the accessory flagellum is composed of five articles in Korean specimens (vs. six articles in Japanese specimens), 2) the large teeth on medial edges of incisors are absent in Korean specimens (vs. two and one tooth on left and right mandibles, respectively in Japanese specimens), and 3) there are eight robust setae on the apical margin of the palp of maxilla 1 (vs. six setae in Japanese specimens).

####### Anonyx exilipes sp. n.

Taxon classificationAnimaliaAmphipodaUristidae

http://zoobank.org/F41912F7-0DA7-435C-A75C-FD6A841EB753

Figs 5 , 6 , 7 , 8

######## Type locality.

Near Daejin Port, Daejin-ri Hyeonnae-myeon Goseong-gun Gangwon-do South Korea. The specimens were collected from fishery nets of this port. According to the statements of fishermen, these nets were brought out within a 5 km radius from Daejin Port and the nets were deployed in about 1–2 km depth (The precise coordinates were uncertain).

######## Material examined.

**Holotype**: Male (23.0 mm), NIBRIV0000806537, **paratypes**: two males and two females (18.5 mm–24.9 mm), NIBRIV0000807160; 11 Mar 2016, by TW Jung.

######## Etymology.

The composite epithet of the specific name, *exilipes*, is a combination of the Latin *exilis* and *pes.* This name means ‘slender foot’ referring to the slender shapes of pereopods 6 and 7 compared to those of other species of the *Anonyx laticoxae* group.

######## Diagnosis.

Gnathopod 1 basis with setae along entire anterior margin; palm serrated; dactylus without protrusion. Gnathopod 2 propodus half as long as carpus, posterodistal corner produced distally, palm short, with small cavity; dactylus short, apex not exceeding corner of palm. Pereopods 3–4 each propodus with single locking setae posterodistally. Pereopod 6 carpus and propodus elongate, slender. Pereopod 7 merus not lobate. Epimeron 2 posteroventral corner acutely produced backwards. Epimeron 3 regularly rounded posteroventrally, posterior margin produced backwards. Uropod 2 both rami without constrictions. Uropod 3 inner ramus as long as proximal article of outer ramus.

######## Description of holotype male.

**Figure 5.**
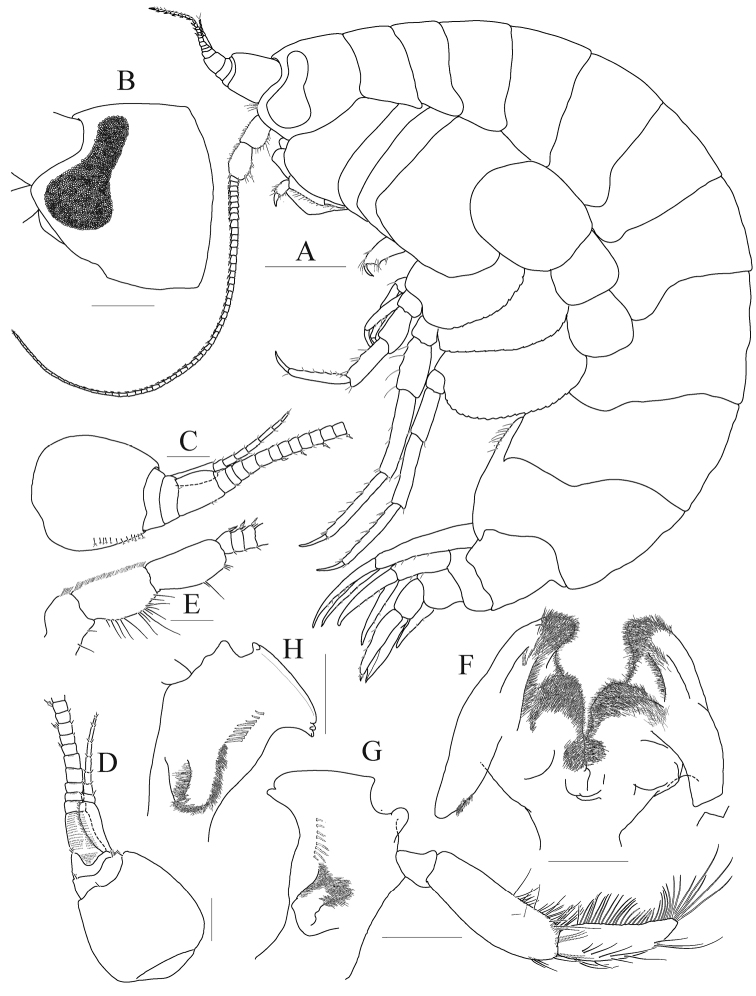
*Anonyx exilipes* sp. n. holotype, male, NIBRIV0000806537, 23.0 mm. **A** habitus **B** head **C** antenna 1, lateral **D** antenna 1, medial **E** antenna 2 peduncular articles **F** lower lip **G** right mandible **H** left mandible. Scale bars: 0.5 mm (**C–H**), 1.0 mm (**B**), 2.0 mm (**A**)

***Head*** (Fig. 5B). Lateral cephalic lobes expanded anteriorly, subtriangular, apex rounded; eye large, pyriform, occupying most of anterior part of head, composed of numerous small ommatidia.

*Antenna 1* (Fig. 5C, D) distinctly shorter than antenna 2; peduncle 1^st^ article ovoid, expanded; 2^nd^ and 3^rd^ articles reduced; accessory flagellum composed of eight articles, 1^st^ article longest, dilated distally, lined with several clusters of minute setae on posterior margin; flagellum 1^st^ article distinctly elongate, calceoli present from 7^th^ article.

*Antenna 2* (Fig. 5A, E) elongate, 0.4 × as long as body; peduncle 4^th^, 5^th^ articles convex posteriorly; 4^th^ article setose on anterior margin; flagellum composed of 63 articles; calceoli present anterodistally.

*Lower lip* (Fig. 5F) densely pubescent; inner lobe distinct.

*Mandible* (Fig. 5G, H) incisor smooth but bearing blunt denticles on both sides; lacinia mobilis absent on both sides; nine and eight small raker setae on left and right mandibles respectively; molar process not triturative, flap-shaped, densely pubescent, lateral setigerous crest present; palp composed of three articles, attached nearly at level of molar process, 2^nd^ article longest, setose anterodistally, 3^rd^ article falcate 0.8 × as long as 2^nd^ article, lined with setae on inner margin and apex.

**Figure 6.**
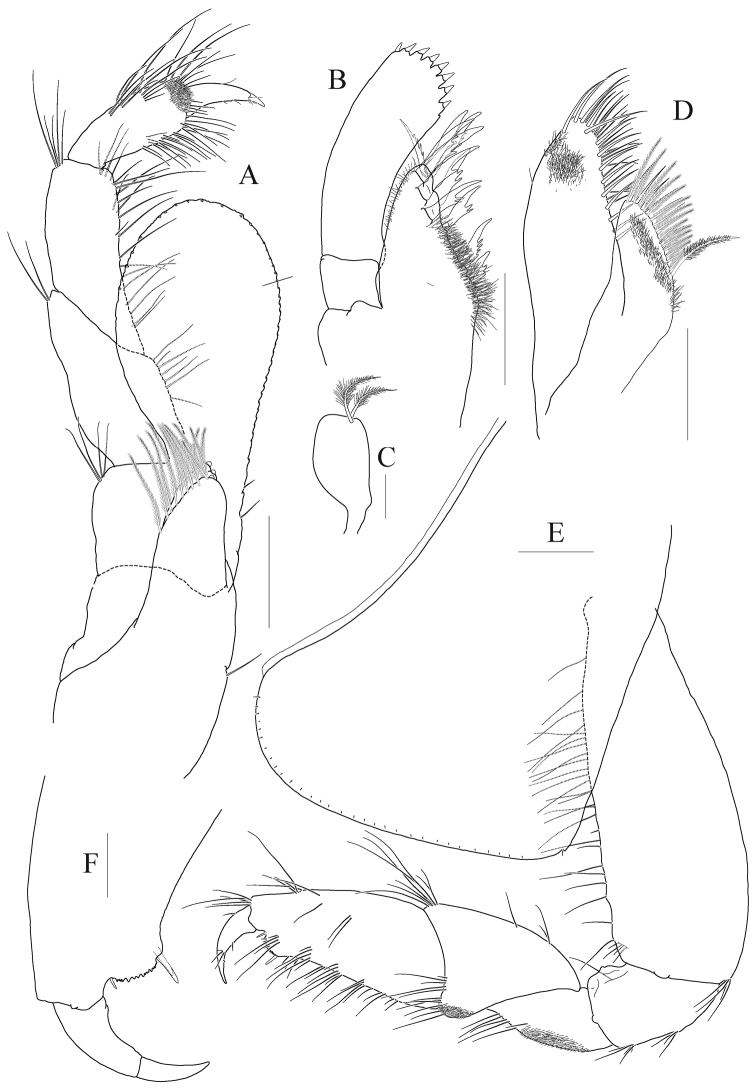
*Anonyx exilipes* sp. n. holotype, male, NIBRIV0000806537, 23.0 mm. **A** maxilliped **B** maxilla 1 **C** maxiila 1 inner lobe **D** maxilla 2 **E** gnathopod 1 **F** gnathopod 1 palm and dactylus. Scale bars: 0.2 mm (**C, F**), 0.5 mm (**A, B, E**).

*Maxilla 1* (Fig. 6B, C) inner lobe short, subquadrate distally, with two plumose setae on blunt apex; outer lobe with eleven toothed setae in 7/4 arrangement; palp composed of two articles, distal article slightly dilated and curved distally, with eight robust setae on apical margin.

*Maxilla 2* (Fig. 6D) inner lobe reduced, half as long as outer lobe, narrowing distally, with two rows of simple and plumose setae on mediodistal margin (proximal plumose seta longest); outer lobe also narrowing distally and with two setal rows on mediodistal margin.

*Maxilliped* (Fig. 6A) inner lobe with mediodistal row of plumose setae, apex rounded with three nodular setae; outer lobe well developed, subovoid, not beyond the palp 3^rd^ article lined with many nodular setae from medial to distal half of lateral margins (all nodular setae small); palp composed of four articles, 2^nd^ article 1.1 × as long as 1^st^ article, with setae medially, 3^rd^ article slightly dilated distally, 0.7 × as long as 2^nd^ article, covered with minute setae distally and with many elongate setae, 4^th^ article 0.7 × as long as 3^rd^ article, apical seta robust, short.

***Pereon.*** *Gnathopod 1* (Fig. 6E, F) subchelate; coxa large, subtrapezoidal, expanded anteroventrally, posteroventral notch nearly weak; basis stout, as long as coxa, anterior margin straight, with setae along entire margin, posterior margin expanded distally, smooth, only with one cluster of setae at distal corner; ischium moderate in size, with one small anterior lobe; merus triangular, 0.3 × as long as basis, covered with minute setae posteriorly; carpus half as long as basis, convex anteroproximally, carpal lobe weak, apex rounded and covered with minute setae; propodus as long as carpus, gradually diminished distally but forming weak lobe together palm posterodistally, palm distinct, convex, serrated, defined by one pair of elongate robust setae; dactylus falcate, exceeding palm, without protrusion on inner margin, unguis developed.

**Figure 7.**
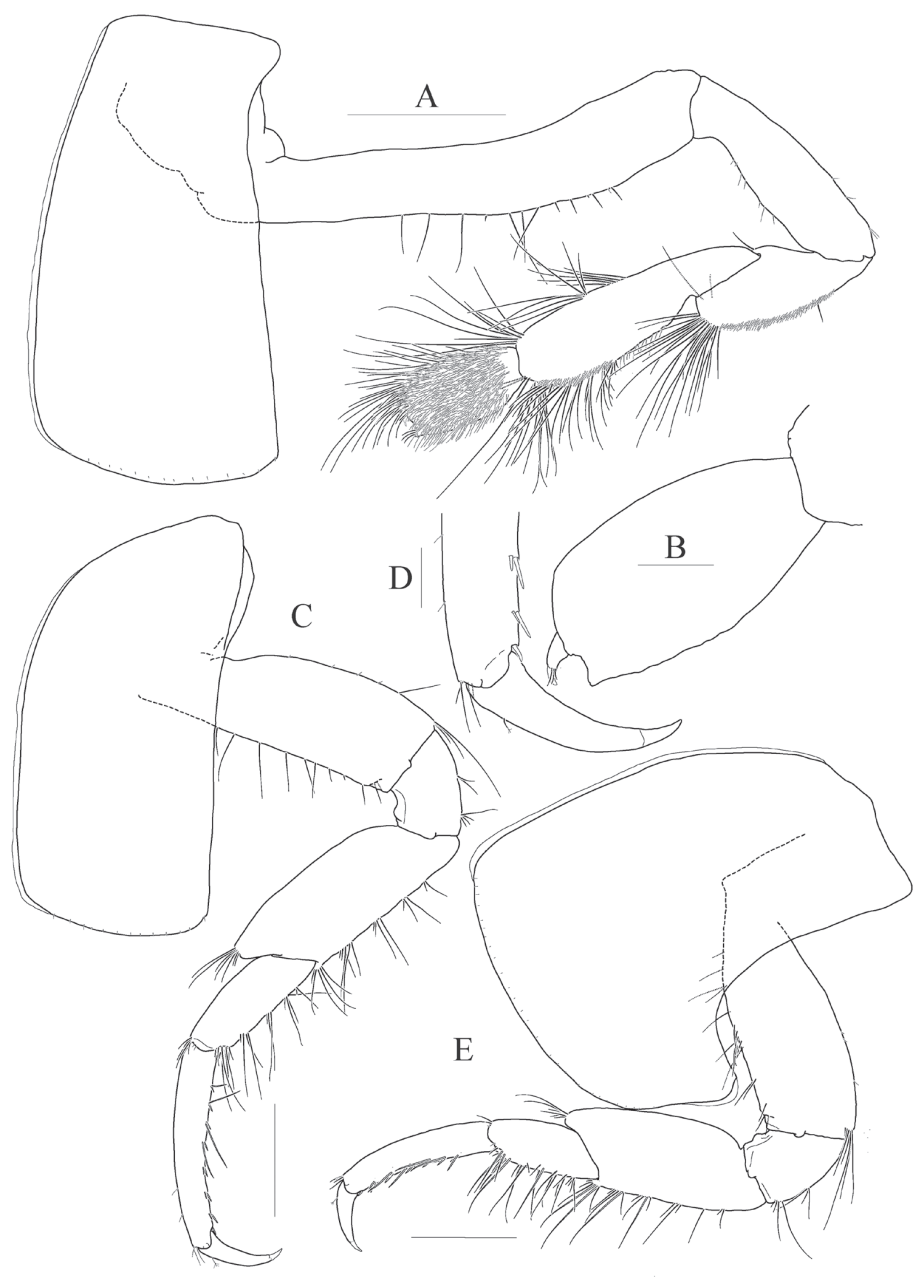
*Anonyx exilipes* sp. n. holotype, male, NIBRIV0000806537, 23.0 mm. **A** gnathopod 2 **B** gnathopod 2 propodus and dactylus, setae omitted **C** pereopod 3 **D** pereopod 3 locking seta and dactylus **E** pereopod 4. Scale bars: 0.2 mm (**B, D**), 1.0 mm (**A, C, E**).

*Gnathopod 2* (Fig. 7A–B) slender, minutely chelate; coxa subrectangular, slightly divergent ventrally, posteroventral notch nearly weak; basis 1.1 × as long as coxa, curved at distal 2/3 length; ischium elongate, half as long as basis; merus 0.8 × as long as ischium, with numerous short setae posteriorly, posterodistal corner angulate with many elongate setae; carpus 0.6 × as long as basis, anterior margin with three clusters of elongate setae on distal half (longest seta of distal cluster exceeding propodus), carpal lobe flattened, distal half margin also with elongate setae and covered with minute setae; propodus subrectangular, half as long as carpus, margins convex, lateral surface densely covered with setae, posterodistal corner produced distally, palm short, with small cavity; dactylus short, anchored at middle of distal margin on propodus, apex not exceeding corner of palm.

*Pereopod 3* (Fig. 7C, D) coxa subrectangular, half as wide as long, posteroventral notch rather weak; basis 0.6 × as long as coxa, anterior margin straight, with eleven setae regularly, posterior margin expanded distally; ischium moderate in size, with one small anterior lobe; merus 0.8 × as long as basis, expanded anteriorly, slightly produced anterodistally; carpus 0.6 × as long as merus, not expanded; propodus 1.7 × as long as carpus, lined with paired setae on posterior margin, with one locking seta posterodistally; dactylus falcate, elongate, 0.4 × as long as propodus, unguis weak.

*Pereopod 4* (Fig. 7E) coxa deeper than wide, expanded posteroventrally; other articles nearly similar with those of pereopod 3.

**Figure 8.**
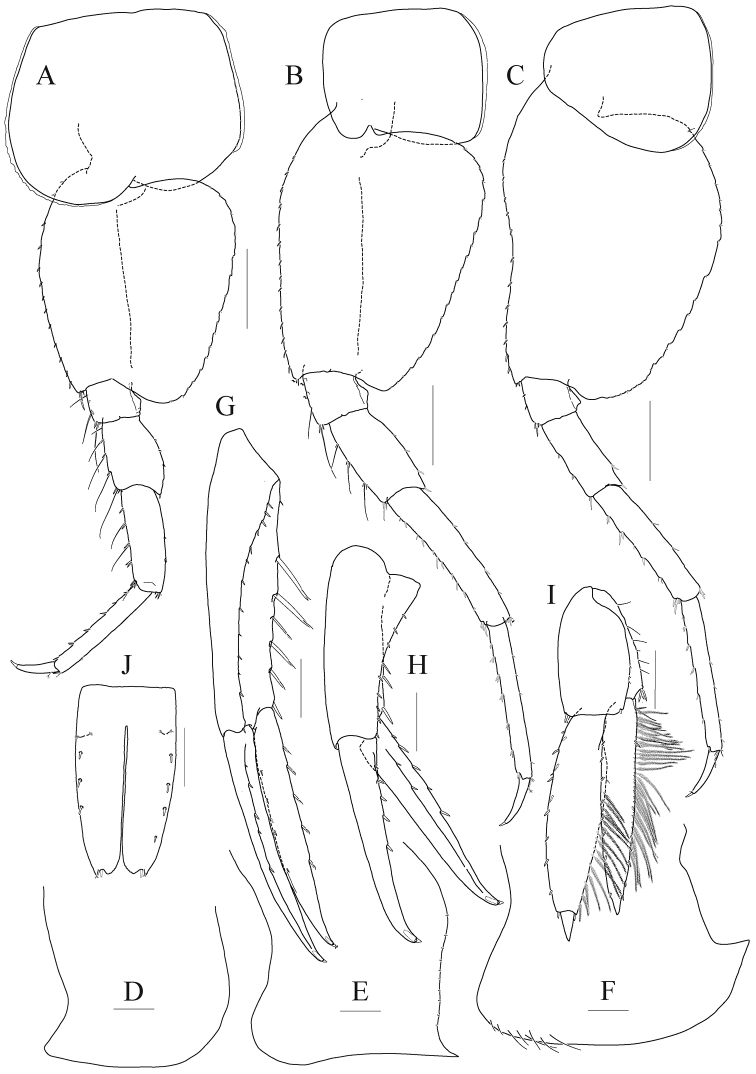
*Anonyx exilipes* sp. n. holotype, male, NIBRIV0000806537, 23.0 mm. **A** pereopod 5 **B** pereopod 6 **C** pereopod 7 **D–F** pleonal epimera 1–3 **G** uropod 1 **H** uropod 2 **I** uropod 3 **J** telson. Scale bars: 0.5 mm (**D–J**), 1.0 mm (**A–C**).

*Pereopod 5* (Fig. 8A) coxa large, subrectangular, 1.2 × as wider as long, equilobate; basis subovoid, anterior margin rounded, lined with robust setae, with one pair of robust setae anterodistally (one seta elongate), posterior lobe well developed, more expanded proximally, margin somewhat flattened, crenulate, expanded posterodistal corner not exceeding ischium; ischium to carpus lined with elongate slender setae and short setae anteriorly; merus posterior lobe expanded distally; carpus subrectangular, 1.3 × as long as merus, posterior margin slightly swollen in midway; propodus linear, 1.1 × as long as carpus, lined with robust setae anteriorly, with one pair of locking setae; dactylus falcate, elongate, 0.4 × as long as propodus, unguis weak.

*Pereopod 6* (Fig. 8B) longest; coxa subrectangular, smaller than that of pereopod 5, bilobate, anterior lobe small, posterior lobe more expanded posteroventrally; basis subovoid, as wide and 1.3 × as long as that of pereopod 5, anterior margin lined with robust setae regularly, posterior lobe well developed, margin somewhat flattened, crenulate, expanded posterodistal corner not angulate, not reaching distal end of ischium; elongate slender setae present from ischium to merus anteriorly; merus subrectangular, 0.4 × as long as basis, slightly expanded posteriorly, weakly produced posterodistally; carpus rectangular, not lobate, 1.6 × as long as merus, a little curved and slightly diminished distally; propodus slender, linear, as long as carpus, lined with robust setae anteriorly, with one pair of locking setae; dactylus falcate, elongate, 0.4 × as long as propodus, unguis weak.

*Pereopod 7* (Fig. 8C) 0.9 × as long as pereopod 6; coxa unilobate, as large as that of pereopod 6, expanded posteroventrally; basis 1.1 × as wide and 1.1 × as long as that of pereopod 6, anterior margin slightly concaved at the middle, lined with robust setae, posterior lobe well developed, margin rounded, not flattened than those of pereopods 6–7; slender setae absent in ischium and merus; merus rectangular, not lobate, 0.3 × as long as basis, weakly produced posterodistally; carpus also not lobate, 1.6 × as long as merus; propodus slender, linear, 1.1 × as long as carpus, lined with robust setae anteriorly, with one pair of locking setae; dactylus falcate, elongate, 0.4 × as long as propodus, unguis weak.

***Pleon.*** *Epimeron 1* (Fig. 8D) weakly produced anteroventrally, rounded posteroventrally. *Epimeron 2* (Fig. 8E) slightly larger than epimeron 1, also produced anteroventrally, slightly convex ventrally, posteroventral corner acutely produced backwards. *Epimeron 3* (Fig. 8F) largest, regularly rounded posteroventrally, posterior margin produced backwards. *Urosomite 1* with deep dorsal depression and distal carina weak (Fig. 5A).

*Uropod 1* (Fig. 8G) longest; peduncle 1.3 × as long as inner ramus, with twelve robust setae on dorsolateral margin and six elongate robust setae on dorsomedial margin; rami subequal to each other; inner ramus with five dorsomedial and four dorsolateral robust setae; outer ramus with five dorsolateral setae and one dorsomedial seta.

*Uropod 2* (Fig. 8H) 0.8 × as long as uropod 1; peduncle as long as inner ramus, with eight robust setae on dorsolateral margin and three robust setae on dorsomedial margin; both rami without constriction; inner ramus with two dorsolateral and five dorsomedial setae; outer ramus as long as inner ramus, with five dorsolateral robust setae.

*Uropod 3* (Fig. 8I) 0.9 × as long as uropod 2; peduncle half as long as inner ramus; both rami with plumose setae on medial margin; outer ramus bi-articulate, distal article 0.2 × as long as proximal article; inner ramus 0.9 × as long as proximal article of outer ramus.

*Telson* (Fig. 8J) longer than broad, cleft to about 80%, each lobe with apical notch bearing one pair of robust seta and sensory seta, three or four robust setae and one pair of sensory setae dorsolaterally.

######## Remarks.

Steele (1986) divided the genus *Anonyx* into five subgroups according to the shapes of uropod 2. Among them, the *Anonyx laticoxae* group is characterized by sharing of the following features: uropod 2 is narrow, its inner ramus unconstricted, and with the distal seta equal to or only slightly longer than the proximal setae. This new species also has this character states and can be included in the *A. laticoxae* group. Moreover, *Anonyx exilipes* sp. n. shares several characteristic features with *Anonyx laticoxae* Gurjanova, 1962 such as similarly produced pleonal epimera, the similar expansions of coxae 1–4, and pereopods 3–4 having single locking setae on their propodus. However, *Anonyx exilipes* sp. n. differs from *A. laticoxae* by the different character states of the carpus and propodus of pereopod 6, which are longer and more slender, and the merus of pereopod 7, which is not lobate in the new species.

## Supplementary Material

XML Treatment for Anonyx abei

XML Treatment for Anonyx exilipes
